# Books and Pamphlets Received

**Published:** 1886-05-20

**Authors:** 


### BOOKS AND PAMPHLETS RECEIVED.

*Physician's Pocket Manual and Year Book* By C. Lowell Austin, M. D. Detroit, Mich : George S. Davis, Medical Publisher. 1881.

We are much pleased with this excellent little work, in which we find presented in a condensed and pithy manner much that is practical and useful on Clinical Diagnosis, Forensic Medicine, Toxicology, Chemical and Microscopical Tests ; Medical and Surgical Memoranda ; Obstetric Procedure ; Weights, Measures and Abbreviations ; Posology, Doses, etc. ; Therapeutical Progress ; Improved Instruments, Albuminoid Phosphates. Under these several heads the practitioner can get a great deal of useful information in a shape readily obtainable.

